# Structure Controlling Factors of Oxido-Bridged Dinuclear Iron(III) Complexes

**DOI:** 10.3390/molecules26040897

**Published:** 2021-02-08

**Authors:** Ryusei Hoshikawa, Kosuke Yoshida, Ryoji Mitsuhashi, Masahiro Mikuriya, Takashi Okuno, Hiroshi Sakiyama

**Affiliations:** 1 Department of Science, Faculty of Science, Yamagata University, 1-4-12 Kojirakawa, Yamagata 990-8560, Japan; hosiryu@yahoo.com (R.H.); thombrowne830@icloud.com (K.Y.); okuno@sci.kj.yamagata-u.ac.jp (T.O.); 2 Institute of Liberal Arts and Science, Kanazawa University, Kakuma, Kanazawa, Ishikawa 920-1192, Japan; mitsuhashi@staff.kanazawa-u.ac.jp; 3 School of Science and Technology, Kwansei Gakuin University, 2-1 Gakuen, Sanda, Hyogo 669-1337, Japan; junpei@kwansei.ac.jp

**Keywords:** oxido-bridged dinuclear iron(III) complex, crystal structure, intramolecular interaction, magnetic properties, density functional theory (DFT)

## Abstract

Oxido bridges commonly form between iron(III) ions, but their bond angles and symmetry vary with the circumstances. A large number of oxido-bridged dinuclear iron(III) complexes have been structurally characterized. Some of them belong to the *C*_2_ point group, possessing bent Fe–O–Fe bonds, while some others belong to the *C_i_* symmetry, possessing the linear Fe–O–Fe bonds. The question in this study is what determines the structures and symmetry of oxido-bridged dinuclear iron(III) complexes. In order to gain further insights, three oxido-bridged dinuclear iron(III) complexes were newly prepared with 2,2′-bipyridine (bpy) and 1,10-phenanthroline (phen) ligands: [Fe_2_OCl_2_(bpy)_4_][PF_6_]_2_ (**1**), [Fe_2_O(NO_3_)_2_(bpy)_4_][PF_6_]_2_·0.6MeCN·0.2(2-PrOH) (**2**), and [Fe_2_OCl_2_(phen)_4_][PF_6_]_2_·MeCN·0.5H_2_O (**3**). The crystal structures of **1**, **2**, and **3** were determined by the single-crystal X-ray diffraction method, and all of them were found to have the bent Fe–O–Fe bonds. Judging from the crystal structure, some intramolecular interligand hydrogen bonds were found to play an important role in fixing the structures. Additional density functional theory (DFT) calculations were conducted, also for a related oxido-bridged dinuclear iron(III) complex with a linear Fe–O–Fe bond. We conclude that the Fe–O–Fe bridge tends to bend like a water molecule, but is often stretched by interligand steric repulsion, and that the structures are mainly controlled by the intramolecular interligand interactions.

## 1. Introduction

The iron(III)–oxido bond is easy to form, and the oxido ligand tends to bridge iron(III) ions. A typical example is a reddish-brown precipitation of hydroxidooxidoiron(III) by mixing the aqueous iron(III) nitrate solution and the sodium hydroxide solution at room temperature. The precipitation has been identified by X-ray diffraction [1] to have the same structure of the natural ore goethite, α-Fe(O)(OH), in which iron(III) ions are bridged by the oxido and hydroxido ligands to form a polymeric structure [2]. The oxido-bridged dinuclear iron(III) motif is also often observed in various coordination compounds in biological systems. The oxy-form of hemerythrin has a (μ-oxido)bis(μ-carboxylato)diiron(III) unit at the active site [3], and the two-electron-reduced dioxygen species (hydroperoxido ligand) is bound to one of the iron(III) ions and to the bridging oxido ligand. In another example, some (μ-oxido)diiron(III) complexes are known to cause characteristic renal injuries [4].

Various kinds of oxido-bridged dinuclear iron(III) complexes have been synthesized, and their structural and physicochemical properties have been studied [5,6,7,8,9,10,11,12,13,14,15,16]. In the case of symmetric dinuclear iron(III) complexes, some belong to the *C*_2_ point group, possessing the twofold axis (*C*_2_ axis) through each oxido ligand, and some others belong to the *C_i_* point group, possessing the inversion center at each oxido ligand. From the magnetic point of view, the Dzyaloshinskii–Moriya interaction [17,18] is expected for the *C*_2_ symmetry, but not for the *C_i_* symmetry. Therefore, it is important to control the symmetry of metal complexes. In this study, three dinuclear iron(III) complexes were newly prepared with rigid 2,2′-bipyridine (bpy) and 1,10-phenanthroline (phen) ligands for the purpose of clarifying the factors in controlling the structures.

## 2. Results and Discussion

### 2.1. Preparation of Complexes, **1**, **2**, and **3**

Three dinuclear iron(III) complexes, [Fe_2_OCl_2_(bpy)_4_][PF_6_]_2_ (**1**), [Fe_2_O(NO_3_)_2_(bpy)_4_][PF_6_]_2_·0.6MeCN·0.2(2-PrOH) (**2**), and [Fe_2_OCl_2_(phen)_4_][PF_6_]_2_·MeCN·0.5H_2_O (**3**), were newly prepared in this study. Complexes **1**, **2**, and **3** were all prepared in the dark to prevent photoreaction that results from the reduced species (e.g., [Fe(bpy)_3_][PF_6_]_2_ [19]). In both IR spectra of **1** and **2** (Figures S1 and S2), the two strong bands were observed at around 1445 and 1600 cm^–1^ and were assigned to the ring stretching of the bpy moiety, where the corresponding bands were observed at around 1415–1455 cm^–1^ and 1560–1580 cm^–1^ for the free bpy ligand. On the other hand, for **3** (Figure S3), the strong bands at around 1425 and 1520 cm^–1^ were assigned to the ring stretching of the phen moiety, and the corresponding bands were observed at around 1410–1425 cm^–1^ and 1495–1505 cm^–1^ for the free phen ligand. Comparing the IR spectra of **1** and **2**, the characteristic intense bands for **2** at around 1390 and 1280 cm^–1^ could be assigned to the monodentate nitrato ligand. The intense band at around 840 cm^–1^ common to complexes **1**–**3** was typical for the hexafluoridophosphate anion. All of the complexes were characterized by elemental analysis and a single-crystal X-ray diffraction study.

### 2.2. Crystal Structures of Complexes, **1**, **2**, and **3**

#### 2.2.1. Crystal Structures of [Fe_2_OCl_2_(bpy)_4_][PF_6_]_2_ (**1**)

**Figure 1:**
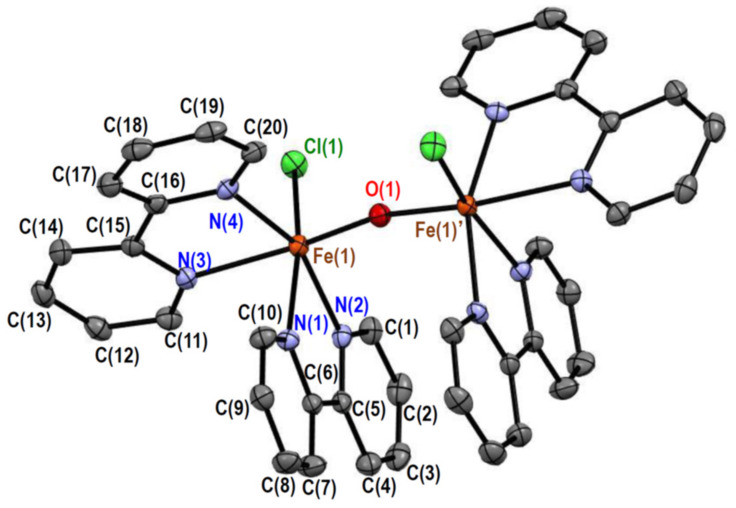
Crystal structure of [Fe_2_OCl_2_(bpy)_4_]^2+^ in **1**. Hydrogen atoms are omitted for clarity. Symmetry code: ′(1 − *x*, *y*, 3/2 − *z*).

The crystal of **1** consists of [Fe_2_OCl_2_(bpy)_4_]^2+^ complex cations and hexafluoridophosphate anions in a 1:2 molar ratio, and no solvent molecules are cocrystallized. The structure of the complex cation is depicted in Figure 1, and its selected bond distances and angles are summarized in Table 1 and Table 2, respectively. The complex cation has a crystallographic twofold axis going through the oxido ligand, bridging the two iron(III) ions. Each iron(III) ion is coordinated by two bipyridine ligands, one chloride ligand, and the one oxido ligand, forming a distorted octahedral ClN_4_O coordination geometry. The average bond angle around the iron(III) ions was 89.7° for the cis-positions, with a standard deviation of 8.9°. The crystal structure of the same complex cation was earlier reported as perchlorate salt, [Fe_2_OCl_2_(bpy)_4_][ClO_4_]_2_·0.25MeCN·0.25MeOH·0.25H_2_O (**4**) [6], and the bond distances and angles around the central iron(III) ions were very similar to each other.

In complex **1** (Figure 1), two bpy moieties, bound to different iron(III) ions, look stacked; however, no attractive interaction is expected because the average separation [3.595(2) Å] was larger than the sum of the van der Waals radius of two carbon atoms (3.4 Å). On the contrary, an intramolecular CH···O hydrogen bond was observed between a bpy moiety and the bridging oxido ligand. Both the C(20)···O(1) [3.043(2) Å] and H(20)···O(1) (~2.55 Å) distances were shorter than the sum of the corresponding van der Waals radii, 3.22 and 2.61 Å, respectively. In addition, an intramolecular CH···Cl hydrogen bond was observed, judging from the Cl(1)···H(20)’ distance (~2.62 Å), shorter than the sum of the van der Waals radii (2.84 Å).

#### 2.2.2. Crystal Structures of [Fe_2_O(NO_3_)_2_(bpy)_4_][PF_6_]_2_·0.6MeCN·0.2(2-PrOH) (**2**)

**Figure 2:**
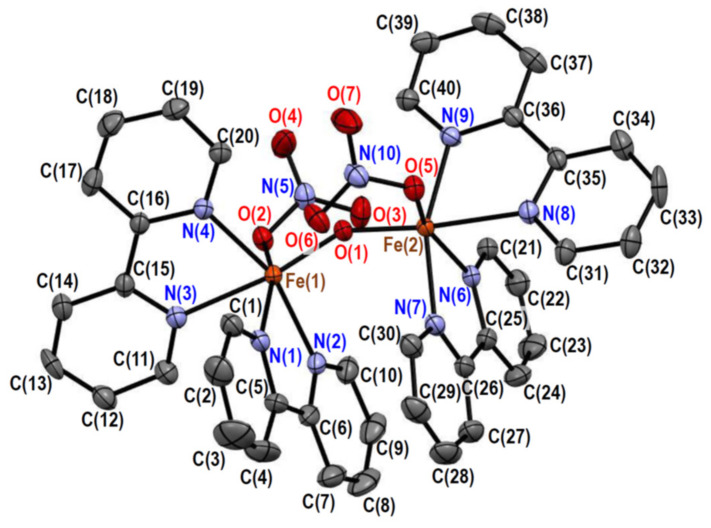
Crystal structure of [Fe_2_O(NO_3_)_2_(bpy)_4_]^2+^ in **2**. Hydrogen atoms are omitted for clarity.

The crystal of **2** consists of [Fe_2_O(NO_3_)_2_(bpy)_4_]^2+^ complex cations, hexafluoridophosphate anions, acetonitrile molecules, and 2-propanol molecules in a 1:2:0.6:0.2 molar ratio. The structure of the complex cation is depicted in Figure 2, and its selected bond distances and angles are summarized in Table 3 and Table 4, respectively. The complex cation has a pseudo-twofold axis going through the oxido ligand, bridging the two iron(III) ions. Each iron(III) ion is coordinated by two bipyridine ligands, one monodentate nitrato ligand, and the one oxido ligand, forming a distorted octahedral N_4_O_2_ coordination geometry. The average bond angle around the iron(III) ions was 89.6° for the cis-positions, with a standard deviation of 9.5°, which was slightly larger than that of **1**. This indicates that the octahedral coordination geometry of **2** is slightly more distorted than that of **1**. Although the crystal structures of the chlorido derivative (**4**) and the sulfato derivative, [Fe_2_O(SO_4_)_2_(bpy)_4_]·11H_2_O (**5**) [10], have been reported, the structure of the nitrato derivative seems to be new. The bond distances and angles around the central iron(III) ions in **2** were not so different from those of the derivatives **1**, **4**, and **5**. The average bond angle around the iron(III) ions in **5** was 89.8° for the cis-positions, with a standard deviation of 9.1°, indicating that the coordination geometries are more distorted with the larger nitrato and sulfato ligands than with the smaller chloride ligand.

In complex **2** (Figure 2), a π–π stacking was observed between two bpy moieties, which was evidenced by the average plane separation [3.351(4) Å] shorter than the C···C van der Waals distance (3.4 Å). Like in **1**, an intramolecular CH···O hydrogen bond was observed between a bpy moiety and the bridging oxido ligand. The C(20)···O(1) [3.107(3) Å] and C(40)···O(1) [3.107(3) Å] distances were shorter than the C···O van der Waals distances (3.22 Å), and the H(20)···O(1) (2.58 Å) and H(40)···O(1) (2.58 Å) distances were shorter than the H···O van der Waals distances (2.61 Å). In **2**, another attractive interaction was the CH···O hydrogen bond between the bpy and nitrato moieties. The H···O distances [H(10)···O(3): 2.50 Å; H(30)···O(6): 2.52 Å] were shorter than the H···O van der Waals distance (2.61 Å).

#### 2.2.3. Crystal Structures of [Fe_2_OCl_2_(phen)_4_][PF_6_]_2_·MeCN·0.5H_2_O (**3**)

**Figure 3:**
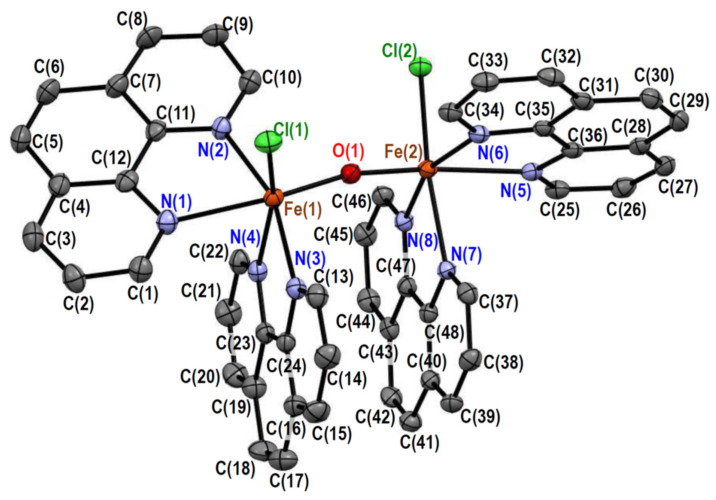
Crystal structure of [Fe_2_OCl_2_(phen)_4_]^2+^ in **3**. Hydrogen atoms are omitted for clarity.

The crystal of **3** consists of [Fe_2_OCl_2_(phen)_4_]^2+^ complex cations, hexafluoridophosphate anions, acetonitrile molecules, and water molecules in a 1:2:1:0.5 molar ratio. The structure of the complex cation is depicted in Figure 3, and its selected bond distances and angles are summarized in Table 5 and Table 6, respectively. The complex cation has a pseudo-twofold axis going through the oxido ligand, bridging the two iron(III) ions. Each iron(III) ion is coordinated by two phenanthroline ligands, one chloride ligand, and the one oxido ligand, forming a distorted octahedral ClN_4_O coordination geometry. The average bond angle around the iron(III) ions was 89.7° for the cis-positions, with a standard deviation of 8.5°, which was comparable to that of **1**, but slightly smaller. This indicates that the distortion of the octahedral coordination geometry of **3** is slightly smaller than that of **1**. A crystal structure of an aqua derivative was earlier reported as [Fe_2_O(H_2_O)_2_(bpy)_4_][NO_3_]_4_·5H_2_O (**6**) [13], in which chloride ligands were replaced with aqua ligands. The average Fe–N distance at the cis-positions of the oxido ligand was ~2.17 Å for **3**, which was slightly longer than that for **6** (~2.15 Å), and this is considered to be typical of the high-spin state [13]. From this point of view, the iron(III) ions in both **1** (~2.17 Å) and **2** (~2.14 Å) are considered to be in the high-spin state.

In complex **3** (Figure 3), a π–π stacking was observed between two phen moieties. Although the average plane separation [3.415(5) Å] was comparable to the C···C van der Waals distance (3.4 Å), short C···C distances [e.g., C(17)···C(39) = 3.331(5) Å] were observed. Like in **1** and **2**, an intramolecular CH···O hydrogen bond was observed between a phen moiety and the bridging oxido ligand. The C(10)···O(1) [3.140(5) Å] and C(34)···O(1) [3.072(4) Å] distances were shorter than the C···O van der Waals distance (3.22 Å). In **3**, the CH···Cl hydrogen bond between the phen and chlorido moieties did not seem to be strong.

### 2.3. Magnetic Properties of Complexes, **1**, **2**, and **3**

**Figure 4:**
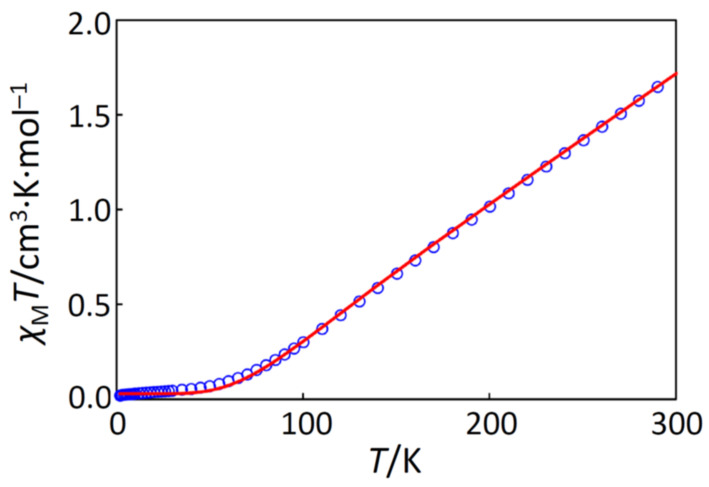
The *χ*_M_*T versus T* plot for **1**. The observed data (○) and the theoretical curve (‒) with the best-fitting parameter set (*J*, *g*, TIP, *ρ*) = (–205 cm^–1^, 2.00, 0 cm^3^·mol^–1^, 0.0017).

The cryomagnetic behaviors for complexes **1**, **2**, and **3** were quite similar to each other, and the *χ*_M_*T* versus *T* plots for **1**, **2**, and **3** are shown in Figure 4 and Figures S4 and S5, respectively. For **1**, the observed *χ*_M_*T* value at 300 K was 0.860 cm^3^·K·mol^–1^, and the *χ*_M_*T* product linearly decreased on cooling to ~80 K (0.089 cm^3^·K·mol^–1^), suggesting a strong antiferromagnetic interaction between the two iron(III) centers. Actually, the magnetic data in the temperature range of 1.9–300 K could be fitted in both high- and low-spin states; however, the magnetic similarity of **1**, **2**, and **3** can be reasonably explained by assuming the high-spin state. If in the low-spin state, the ground term was ^2^*T*_2_, possessing the orbital angular momentum, and the magnetic behavior must be sensitive to the symmetry around the iron(III) ion due to the spin-orbit coupling; the low-temperature data could be fitted with a similar large anisotropic interaction, which is not reasonable. On the contrary, if in the high-spin state, the ground term was ^6^*A*_1_, which is less sensitive to the symmetry, and the low-temperature data were successfully interpreted by a very small amount of paramagnetic impurity, *ρ*. Therefore, the magnetic simulation was conducted with the following equation (Equation (1)) with *x* = *J*/(*kT*), assuming the isotropic exchange interaction with the Hamiltonian **H** = −*J* **S**_1_·**S**_2_ (*S*_1_ = *S*_2_ = 5/2), where *ρ* is the paramagnetic impurity with *S* = 5/2.

(1) $$\chi_{M}=2\left(\frac{Ng^{2}\beta^{2}}{kT}\frac{e^{x}+5e^{3x}+14e^{6x}+30e^{10x}+55e^{15x}}{1+3e^{x}+5e^{3x}+7e^{6x}+9e^{10x}+11e^{15x}}+\mathrm{TIP}\right)\left(1-\rho \right)+2\left(\frac{35Ng^{2}\beta^{2}}{12kT}+\mathrm{TIP}\right)\rho$$

The best-fitting parameter set was obtained as (*J*, *g*, TIP, *ρ*) = (–205 cm^–1^, 2.00, 0 cm^3^·mol^–1^, 0.0017) with a good discrepancy factor of *R*(*χ*_M_*T*) = 2.8 × 10^–4^. In the data fitting, the *g*-factor and TIP (temperature-independent paramagnetism) were fixed to 2.00 and 0 cm^3^·mol^–1^, respectively, because the iron(III) center has the high-spin *d*^5^ electronic configuration. In the same way, the cryomagnetic data for **2** and **3** were successfully fitted with similar magnetic parameters as summarized in Table 7.

**Figure 5:**
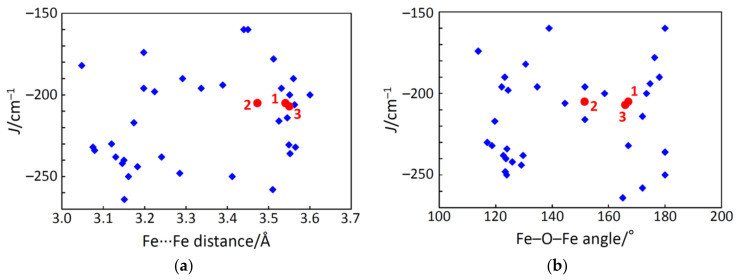
Plots of interaction parameter *J versus* Fe···Fe distances (**a**) and *versus* Fe–O–Fe angles (**b**) for complexes **1**–**3** (●) and related oxido-bridged dinuclear iron(III) complexes (♦).

The obtained magnetic interaction parameters, *J*, were very similar to each other for complexes **1**, **2**, and **3**. With the intension of finding a magnetostructural correlation, the *J* values were plotted against the Fe···Fe distances and the Fe–O–Fe angles with other oxido-bridged dinuclear iron(III) complexes [6,20] (Figure 5). It is noted that the reported values using the different Hamiltonian were corrected for the Hamiltonian **H** = −*J* **S**_1_·**S**_2_. The *J* values were found to fall in the range of −160–−265 cm^–1^ for the oxido-bridged dinuclear iron(III) complexes. In addition, their Fe···Fe distances and Fe–O–Fe angles fell in the ranges of 3.04–3.60 Å and 113–180°, respectively. Although the typical ranges were found for the oxido-bridged dinuclear iron(III) complexes, further correlation was not found.

Since the symmetry of the complex cations in **1**, **2**, and **3** was *C*_2_ or *pseudo*-*C*_2_, the Dzyaloshinskii–Moriya interaction was expected to occur; however, we concluded that the Dzyaloshinskii–Moriya interaction was negligible in the magnetic data of **1**, **2**, and **3** obtained in this study. This may be due to the isotropic ^6^*A*_1_ ground state.

### 2.4. Electronic Spectra of Complexes, **1**, **2**, and **3**

**Figure 6:**
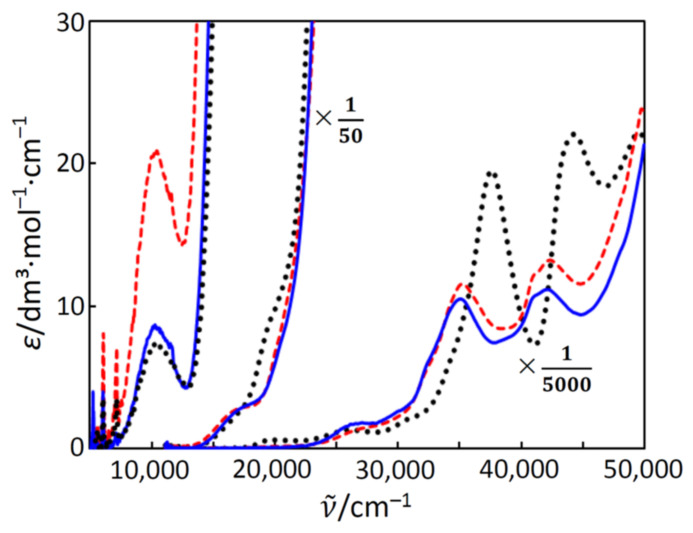
Electronic spectra of **1** (**—**), **2** (---), and **3** (**···**) in acetonitrile.

The electronic spectra of complexes **1**, **2**, and **3** were measured in acetonitrile. Judging from the molar conductance in acetonitrile (see Section 3.3), the complexes were found to act as 2:1 electrolytes in acetonitrile [21], indicating that the dinuclear iron(III) units are stable in acetonitrile. The spectra are shown in Figure 6, and the analyzed spectral components are summarized in Table 8. Each complex shows a weak band in the near-infra-red region, several bands in the visible region, and two intense bands in the near-ultraviolet region. These features were similar to those reported for the related oxido-bridged dinuclear iron(III) complexes [14]. If complexes **1**, **2**, and **3** were compared, **1** and **3** were similar below 20,000 cm^–1^, while **1** and **2** were similar above 20,000 cm^–1^. Remembering the structural features, both **1** and **3** have the ClN_4_O coordination geometry, but **2** has the N_4_O_2_ coordination geometry. On the other hand, **1** and **2** have the bpy ligands, but **3** has the phen ligands. Judging from the spectral similarity between **1** and **3**, possessing the same ClN_4_O coordination geometry, the bands at around 10,000 cm^–1^ (components 1 and 2 in Table 8) are related to the coordination geometries around the iron(III) ions. Since the band intensity of **2** was larger than the others, the ligand-field symmetry around the iron(III) ion in **2** is expected to be lower than the others. That is, the intensity of the Laporte-forbidden d-d band becomes stronger when the symmetry lowers to make the Laporte forbidden relaxed. This is consistent with the larger distortion of **2** than those of the others, judging from the crystal structures (see Section 2.2). The bands in the range of 17,000–25,000 cm^–1^ are common to **1**, **2**, **3**, and other related oxido-bridged iron(III) compounds, and bands are related to the typical red color of the oxido–iron(III) bonds. Above 30,000 cm^–1^, since **1** and **2** were similar, the bands are expected to be more related to the bidentate ligands.

In order to assign the observed components in electronic spectra, spectral simulation was conducted on the basis of the ligand field theory using the angular overlap model (AOM) [22]. For each complex, spectral components 1, 2, and 3 were simulated for the high-spin (*S* = 5/2) state and for the low-spin (*S* = 1/2) state, assuming the ideal *O* symmetry, and the results are summarized in Table 9 and Table 10, respectively. Using the Racah parameters, *B* and *C*, and the average AOM parameter, *e_σ_*_,av_, the spectral components were simulated in both spin states; however, the obtained Racah parameters were abnormal in the low-spin state, especially for complex **2**. Assuming the high-spin state, all of the obtained parameters were normal, and *B* and *C* fell in the ranges of 78%–94% and 70%–83% of the free-ion values (1029 and 4200 cm^–1^), respectively. According to the simulation, components 1 and 2 were assigned to ^6^*A*_1_ → ^2^*T*_2_ and ^6^*A*_1_ → ^4^*T*_1_ bands, respectively, and these assignments were consistent with the earlier studies on μ-oxido-μ-carboxylatodiiron(III) complexes [23,24].

The intensity of spectral components 3 and 4 in Table 8 was stronger than those of the standard d-d transition bands, but as suggested earlier [14], the intensity of the components is considered to be enhanced by the energetically close strong charge-transfer band(s). Based on the simulation in this study, components 3 and 4 were considered to be based on the ^6^*A*_1_ → ^4^*T*_1_ band typically enhanced by the oxido ligand, which was consistent with earlier studies [23,24]. The absorption bands higher in energy could not be assigned in detail using the ideal octahedral model.

### 2.5. Factors in Controlling the Structures

Complex cations in **1**, **2**, and **3** were *C*_2_ or *pseudo*-*C*_2_ symmetry, possessing the bent Fe–O–Fe bridge. However, some of the oxido-bridged dinuclear iron(III) complexes are centrosymmetric (*C_i_* symmetry), possessing the linear Fe–O–Fe bridge [5,7,8]. To avoid the intramolecular steric repulsions between ligands, the centrosymmetric structures are generally preferable; on the other hand, if the two protons of water molecules are replaced with iron(III) ions, the bent bridging structures are considered to be natural. In order to find the factors for controlling the structures, we examined the effects of intramolecular interactions in this section.

**Figure 7:**
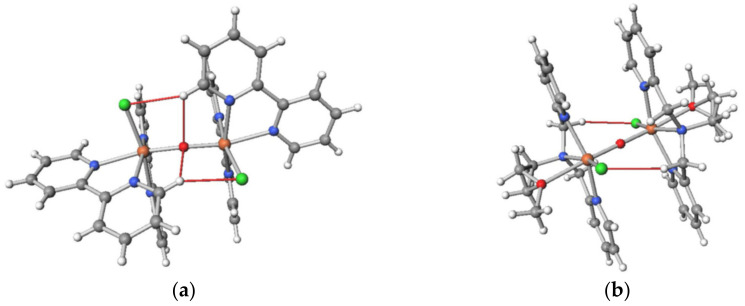
Intramolecular interligand hydrogen bonds in oxido-bridged dinuclear iron(III) complex cations: (**a**) *C*_2_-symmetric [Fe_2_OCl_2_(bpy)_4_]^2+^; (**b**) *C_i_*-symmetric [Fe_2_OCl_2_(epy)_4_]^2+^. Significant hydrogen bonds are shown in red.

In the *C*_2_-symmetric complex cations, the intramolecular CH···O hydrogen bonds were observed between the bpy and oxido ligands. In addition, the intramolecular CH···Cl or CH···O hydrogen bonds were observed between the bpy (or phen) moiety on an iron(III) ion and the anion ligand moiety on another iron(III) ion. These attractive interactions are helpful in fixing the structure in a certain structure. For example, the intramolecular interligand hydrogen bonds are shown for [Fe_2_OCl_2_(bpy)_4_]^2+^ in Figure 7a. The dihedral angles of Cl–Fe···Fe–Cl were 107° in **1** and 79° in **3**, and the dihedral angle of O(nitrato)–Fe···Fe–O(nitrato) was 131°. In each structure, the dihedral angle seems to be fixed to the most stable structure balancing the intramolecular interactions and the intramolecular steric repulsions. In order to check this, for example, energy calculation was conducted on the basis of the density functional theory (DFT) with respect to the dihedral angles in **1**.

**Figure 8:**
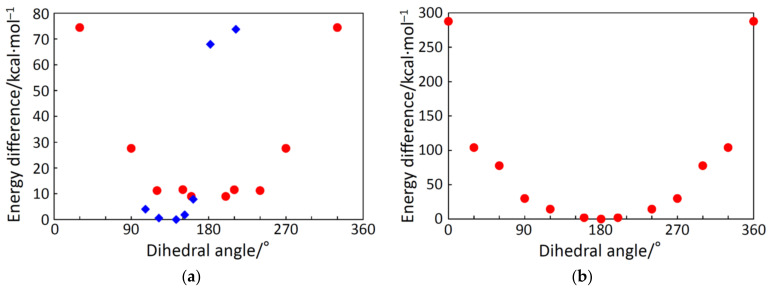
Energy change with respect to the Cl–Fe···Fe–Cl dihedral angle for [Fe_2_OCl_2_(bpy)_4_]^2+^ (**a**) and [Fe_2_OCl_2_(epy)_4_]^2+^ (**b**) on the basis of the *C_i_* structure (●) and the *C*_2_ structure (♦).

The resulting energy values for [Fe_2_OCl_2_(bpy)_4_]^2+^ in **1** were plotted as the energy differences from the most stable energy value in Figure 8a on the basis of the *C_i_* structure and of the *C*_2_ structure. The energy minimum for the *C*_2_ structure was found to be more stable than that for the *C_i_* structure. The energy minimum was observed at around a dihedral angle of ~130°, which was slightly larger than that in the crystal structure (107°). This difference is thought to be caused by the intermolecular interactions, including the CH···Cl interaction between the cations and the CH···F interaction between the cation and anion, observed in the crystal structure. Anyway, the most important factor in controlling the structure was found to be the intramolecular interligand interactions.

Another calculation example was the *C_i_*-symmetric oxido-bridged dinuclear iron(III) complex cation with a tetradentate ligand, *N*-(2-methoxyethyl)-*N*,*N*-bis(pyridin-2-ylmethyl)amine (epy), [Fe_2_OCl_2_(epy)_2_]^2+^ [5,7] (Figure 7b), and the angle dependency is shown in Figure 8b. The structure of this cation was centrosymmetric, and the dihedral angle of Cl–Fe···Fe–Cl was 180°. In the crystal structure, the apparent CH···Cl hydrogen bonds exist between the methylene chain and the chloride ligand, and the hydrogen bonds seem to stabilize the centrosymmetric structure (Figure 7b). In the dihedral angle rotation calculation, a dihedral angle of 180° was found to be the most stable, which was consistent with the crystal structure. Furthermore, other conformations looked no longer preferable due to the interligand steric repulsion. Therefore, the most important factor in controlling the structure was again found to be the intramolecular interligand interactions.

**Figure 9:**
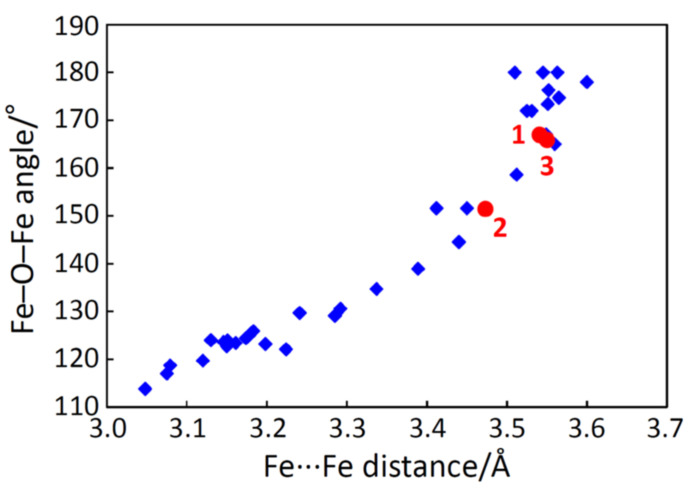
Plots of Fe–O–Fe angles versus Fe···Fe distances for complexes **1**–**3** (●) and related oxido-bridged dinuclear iron(III) complexes (♦).

In complexes **1**, **2**, and **3**, the Fe···Fe separations [3.4729(5)–3.5501(7) Å] cannot be so smaller than the π–π contact, discussed in Section 2.2, judging from the structures. This is a strict barrier caused by the steric requirement. In natural ores, goethite [2] and hematite [25], the effect of the interligand steric repulsion is much smaller because of their small sizes, and their Fe···Fe separations are smaller [~3.01 Å and ~2.90 Å, respectively] as expected. Therefore, we can conclude that the Fe–O–Fe bridge tends to bend like a water molecule, but is often stretched by interligand steric repulsion. A good correlation was observed between the Fe···Fe distance and Fe–O–Fe angle, as shown in Figure 9.

## 3. Materials and Methods

### 3.1. Measurements

Elemental analyses (C, H, and N) were performed at the Elemental Analysis Service Centre of Kyushu University. Iron(III) ions were quantified by titration with ethylenediaminetetraacetic acid in the presence of hydrochloric acid, using variamine blue B as an indicator. IR spectra were recorded on a Jasco FT/IR-4100 FT-IR spectrometer. Electronic spectra were measured at room temperature on Jasco V-560 (200–800 nm) and Hitachi 330 (800–2000 nm) spectrophotometers. Molar conductances were measured in MeOH on a DKK AOL-10 conductivity meter at room temperature. Magnetic susceptibility measurements were performed with a Quantum Design MPMS-7 SQUID magnetometer in the temperature range from 1.9 to 300 K with a static field of 5 kOe. The polycrystalline samples were ground into fine powders in an agate mortar. The sample was wrapped with aluminum foil for **1** and **2**. The sample for **3** was loaded into a gelatin capsule. All data were corrected for paramagnetism of the aluminum foil or diamagnetism of the capsule. The susceptibilities were corrected for the diamagnetism of the samples by means of Pascal’s constants.

### 3.2. Materials

All the chemicals were commercial products and were used as supplied. Methanol, iron(III) nitrate–water (1/9), iron(III) chloride–water (1/6), 2,2′-bipyridine, acetonitrile, 2-propanol, 1,10-phenanthroline, ethylenediaminetetraacetic acid, and hydrochloric acid were supplied by Nacalai Tesque Inc. Sodium hexafluoridophosphate and variamine blue B were supplied by FUJIFILM Wako Pure Chemical Corporation.

### 3.3. Preparations

[Fe_2_OCl_2_(bpy)_4_][PF_6_]_2_ **1**. All operations were conducted in the dark. To a methanolic solution (10 mL) of iron(III) nitrate–water (1/9) (0.54 g, 1.3 mmol) was added a methanolic solution (5 mL) of iron(III) chloride–water (1/6) (0.18 g, 0.66 mmol) and a methanolic solution (5 mL) of 2,2′-bipyridine (0.62 g, 4.0 mmol), and the resulting solution was stirred for 120 min to give a dark-yellow solution. The addition of sodium hexafluoridophosphate (0.40 g, 2.4 mmol) resulted in the precipitation of dark-yellow powder. Recrystallized from acetonitrile/2-propanol to give dark-yellow powder. Yield: 0.40 g (36%) (found: C, 42.90; H, 3.10; N, 10.20; Fe, 10.00; calc. for C_40_H_32_Cl_2_F_12_Fe_2_N_8_OP_2_: C, 43.15; H, 2.90; N, 10.05; Fe, 10.05). Selected IR data [ṽ/cm^–1^] using KBr disk: 3130–3025, 1600, 1445, 1315, 1160, 1025, 840, 770, 560. Molar conductance in MeCN [Λ/S cm^2^·mol^–1^] 232.

[Fe_2_O(NO_3_)_2_(bpy)_4_][PF_6_]_2_·0.6MeCN·0.2(2-PrOH) **2**. All operations were conducted in the dark. To a methanolic solution (10 mL) of iron(III) nitrate–water (1/9) (0.81 g, 2.0 mmol) was added a methanolic solution (5 mL) of 2,2′-bipyridine (0.62 g, 4.0 mmol), and the resulting solution was stirred for 120 min to give a dark-brown solution. The addition of sodium hexafluoridophosphate (0.40 g, 2.4 mmol) resulted in the precipitation of dark-brown powder. Recrystallized from acetonitrile/2-propanol to give dark-brown powder. Yield: 0.60 g (52%) (found: C, 41.85; H, 2.95; N, 12.45; Fe, 9.10; calc. for C_41.8_H_35.4_F_12_Fe_2_N_10.6_O_7.2_P_2_: C, 41.75; H, 2.95; N, 12.35; Fe, 9.30). Selected IR data [*ṽ*/cm^–1^] using KBr disk: 3170–3010, 1600, 1445, 1380, 1280, 1165, 1030, 840, 770, 560. Molar conductance in MeCN [*Λ*/S cm^2^·mol^–1^] 244.

[Fe_2_OCl_2_(phen)_4_][PF_6_]_2_·MeCN·0.5H_2_O **3.** All operations were conducted in the dark. To a methanolic solution (10 mL) of iron(III) chloride–water (1/6) (0.27 g, 1.0 mmol) was added a methanolic solution (5 mL) of 1,10-phenanthroline (0.40 g, 2.0 mmol), and the resulting solution was stirred for 120 min to give a dark-brown solution. After filtration, the addition of sodium hexafluoridophosphate (0.20 g, 1.2 mmol) resulted in the precipitation of dark-brown powder. Recrystallized from acetonitrile/2-propanol to give dark-brown powder. Yield: 0.21 g (35%) (found: C, 47.25; H, 2.80; N, 9.10 Fe, 9.05; calc. for C_48_H_33_Cl_2_F_12_Fe_2_N_8_O_1.5_P_2_ (**3** − MeCN): C, 47.32; H, 2.73; N, 9.20; Fe, 9.15). Selected IR data [ṽ/cm^–1^] using KBr disk: 3165–2980, 1585–1630, 1520, 1150, 1105, 840, 725, 560. Molar conductance in MeCN [Λ/S cm^2^·mol^–1^] 258.

### 3.4. Crystallography

Crystallographic data are summarized in Table 11. Single crystals of **1** and **2** suitable for X-ray analysis were obtained by slow diffusion of 2-propanol to an acetonitrile solution of the complexes. Single crystals of **3** were obtained from an acetonitrile solution of **3**. Single-crystal X-ray diffraction data were obtained with a Bruker SMART APEX or Rigaku XtaLAB AFC11 diffractometer with graphite-monochromated Mo Kα radiation (λ = 0.71073 Å). A single crystal was mounted with a cryoloop or glass capillary and flash-cooled with a cold N_2_ gas stream. Data were processed using the SMART or CrysAlisPro software packages. The structure was solved by intrinsic phasing methods using the SHELXT [26] software packages and refined on *F*^2^ (with all independent reflections) using the SHELXL [27] software packages. The non-hydrogen atoms were refined anisotropically, and hydrogen atoms were refined using the riding model. Complex **2** was refined as a two-component twin. The Cambridge Crystallographic Data Centre (CCDC) deposition numbers are included in Table 11.

### 3.5. Computation

Magnetic analyses and magnetic simulation were conducted using the MagSaki(FeIII)0.0.4 and MagSaki(B)0.7.5 programs of the MagSaki series. AOM calculations were performed using the AOMX program on an Intel Celeron computer. DFT computations were performed using the GAMESS program [28,29] on Fujitsu PRIMERGY CX2550/CX2560 M4 (ITO super computer system) at Kyushu University. Calculations were performed with LC-BOP/6-31G [30].

## 4. Conclusions

Three oxido-bridged dinuclear iron(III) complexes, **1**, **2**, and **3**, were newly prepared, and all of them were found to have the bent Fe–O–Fe bonds. Strong antiferromagnetic interaction was observed for each complex, which is characteristic for the oxido-bridged dinuclear iron(III) complexes. Electronic spectra were examined using the angular overlap model and found to be typical of the oxido-bridged dinuclear iron(III) complexes.

A good correlation was found between the Fe···Fe distance and the Fe–O–Fe angle for the known oxido-bridged dinuclear iron(III) complexes, including **1**–**3**. For the Fe···Fe distance, a strict barrier was found to be caused by the steric requirement of the ligands. From the crystal structure, some intramolecular interligand hydrogen bonds were found to play an important role in controlling the structures.

In this study, we have concluded that the Fe–O–Fe bridge tends to bend like a water molecule, but is often stretched by interligand steric repulsion, and that the structures are mainly controlled by the intramolecular interligand interactions.

## Figures and Tables

**Table 1:** Selected distances for **1**.

Atom–Atom ^1^	Distance/Å	Atom–Atom	Distance/Å
Fe(1)–Cl(1)	2.3119(5)	Fe(1)–O(1)	1.7819(3)
Fe(1)–N(1)	2.2169(15)	Fe(1)–N(2)	2.1446(15)
Fe(1)–N(3)	2.2279(15)	Fe(1)–N(4)	2.1357(15)
Fe(1)···Fe(1)′	3.5407(5)		

^1^ Symmetry code: ′ (1 − *x*, *y*, 3/2 − *z*).

**Table 2:** Selected angles for **1**.

Atom–Atom–Atom	Angle/°	Atom–Atom–Atom	Angle/°
Cl(1)–Fe(1)–O(1)	99.35(4)	Cl(1)–Fe(1)–N(1)	166.69(4)
Cl(1)–Fe(1)–N(2)	97.75(4)	Cl(1)–Fe(1)–N(3)	86.33(4)
Cl(1)–Fe(1)–N(4)	97.71(4)	O(1)–Fe(1)–N(1)	92.71(5)
O(1)–Fe(1)–N(2)	99.01(6)	O(1)–Fe(1)–N(3)	168.39(5)
O(1)–Fe(1)–N(4)	94.71(6)	N(1)–Fe(1)–N(2)	74.65(6)
N(1)–Fe(1)–N(3)	82.83(5)	N(1)–Fe(1)–N(4)	86.81(6)
N(2)–Fe(1)–N(3)	90.17(5)	N(2)–Fe(1)–N(4)	157.33(6)
N(3)–Fe(1)–N(4)	74.40(6)	Fe(1)–O(1)–Fe(1)′	166.95(11)

Symmetry code: ′ (1 − *x*, *y*, 3/2 − *z*).

**Table 3:** Selected distances for **2**.

Atom–Atom	Distance/Å	Atom–Atom	Distance/Å
Fe(1)–O(1)	1.792(19)	Fe(1)–O(2)	2.028(2)
Fe(1)–N(1)	2.167(2)	Fe(1)–N(2)	2.135(3)
Fe(1)–N(3)	2.247(2)	Fe(1)–N(4)	2.131(2)
Fe(2)–O(1)	1.791(19)	Fe(2)–O(5)	2.021(2)
Fe(2)–N(6)	2.166(2)	Fe(2)–N(7)	2.128(2)
Fe(2)–N(8)	2.228(2)	Fe(2)–N(9)	2.120(2)
Fe(1)···Fe(2)	3.4729(5)		

**Table 4:** Selected angles for **2**.

Atom–Atom–Atom	Angle/°	Atom–Atom–Atom	Angle/°
O(1)–Fe(1)–O(2)	99.96(9)	O(1)–Fe(1)–N(1)	101.14(10)
O(1)–Fe(1)–N(2)	100.97(9)	O(1)–Fe(1)–N(3)	170.72(9)
O(1)–Fe(1)–N(4)	96.46(9)	O(2)–Fe(1)–N(1)	158.06(9)
O(2)–Fe(1)–N(2)	94.65(10)	O(2)–Fe(1)–N(3)	80.20(9)
O(2)–Fe(1)–N(4)	91.38(9)	N(1)–Fe(1)–N(2)	75.51(10)
N(1)–Fe(1)–N(3)	79.94(9)	N(1)–Fe(1)–N(4)	92.15(9)
N(2)–Fe(1)–N(3)	88.24(9)	N(2)–Fe(1)–N(4)	160.25(9)
N(3)–Fe(1)–N(4)	74.27(9)	O(1)–Fe(2)–O(5)	101.38(9)
O(1)–Fe(2)–N(6)	98.66(9)	O(1)–Fe(2)–N(7)	98.77(9)
O(1)–Fe(2)–N(8)	172.76(9)	O(1)–Fe(2)–N(9)	98.07(9)
O(5)–Fe(2)–N(6)	159.31(9)	O(5)–Fe(2)–N(7)	95.86(9)
O(5)–Fe(2)–N(8)	80.12(9)	O(5)–Fe(2)–N(9)	92.21(9)
N(6)–Fe(2)–N(7)	75.94(9)	N(6)–Fe(2)–N(8)	80.64(9)
N(6)–Fe(2)–N(9)	90.02(9)	N(7)–Fe(2)–N(8)	88.07(9)
N(7)–Fe(2)–N(9)	159.49(9)	N(8)–Fe(2)–N(9)	74.75(9)
Fe(1)–O(1)–Fe(2)	151.44(12)		

**Table 5:** Selected distances for **3**.

Atom–Atom	Distance/Å	Atom–Atom	Distance/Å
Fe(1)–Cl(1)	2.3315(10)	Fe(1)–O(1)	1.791(2)
Fe(1)–N(1)	2.246(3)	Fe(1)–N(2)	2.134(3)
Fe(1)–N(3)	2.156(3)	Fe(1)–N(4)	2.192(3)
Fe(2)–Cl(2)	2.297(10)	Fe(2)–O(1)	1.786(2)
Fe(2)–N(5)	2.283(3)	Fe(2)–N(6)	2.137(3)
Fe(2)–N(7)	2.212(3)	Fe(2)–N(8)	2.165(3)
Fe(1)···Fe(2)	3.5501(7)		

**Table 6:** Selected angles for **3**.

Atom–Atom–Atom	Angle/°	Atom–Atom–Atom	Angle/°
Cl(1)–Fe(1)–O(1)	100.59(8)	Cl(1)-Fe(1)–N(1)	88.26(7)
Cl(1)–Fe(1)–N(2)	93.74(8)	Cl(1)-Fe(1)–N(3)	94.93(8)
Cl(1)–Fe(1)–N(4)	167.13(8)	O(1)-Fe(1)–N(1)	167.95(11)
O(1)–Fe(1)–N(2)	95.65(11)	O(1)–Fe(1)–N(3)	98.64(11)
O(1)–Fe(1)–N(4)	90.06(10)	N(1)–Fe(1)–N(2)	75.45(11)
N(1)–Fe(1)–N(3)	88.63(10)	N(1)–Fe(1)–N(4)	82.32(10)
N(2)–Fe(1)–N(3)	161.61(11)	N(2)–Fe(1)–N(4)	92.37(10)
N(3)–Fe(1)–N(4)	76.16(10)	Cl(2)–Fe(2)–O(1)	101.91(8)
Cl(2)–Fe(2)–N(5)	86.96(8)	Cl(2)–Fe(2)–N(6)	95.77(9)
Cl(2)–Fe(2)–N(7)	162.27(8)	Cl(2)–Fe(2)–N(8)	93.54(8)
O(1)–Fe(2)–N(5)	166.51(11)	O(1)–Fe(2)–N(6)	94.16(11)
O(1)–Fe(2)–N(7)	94.29(11)	O(1)–Fe(2)–N(8)	102.05(11)
N(5)–Fe(2)–N(6)	74.67(11)	N(5)–Fe(2)–N(7)	78.49(11)
N(5)–Fe(2)–N(8)	87.34(11)	N(6)–Fe(2)–N(7)	90.14(11)
N(6)–Fe(2)–N(8)	159.21(11)	N(7)–Fe(2)–N(8)	75.87(11)
Fe(1)–O(1)–Fe(2)	165.87(15)		

**Table 7:** Magnetic parameters of the present complexes.

Complex	1	2	3
*J*/cm^–1^	–205	–205	–207
*g* (fixed)	2.00	2.00	2.00
TIP/cm^3^·mol^–1^ (fixed)	0	0	0
*ρ*	0.0017	0.0011	0.0035

**Table 8:** Spectral components (cm^–1^) for **1**, **2**, and **3**. ^1^

Component	1	2	3
1	9410 (3.74)	9870 (18.4)	9370 (2.21)
2	11,000 (7.53)	12,100 (11.4)	10,900 (6.78)
3	16,400 (87.9)	16,100 (171)	17,200 (91.5)
4	18,500 (103)	17,800 (179)	19,000 (88.0)
5	21,400 (320)	20,900 (683)	20,200 (438)
6		23,700 (683)	
7	25,800 (3940)	26,300 (2370)	25,300 (5150)
8	30,100 (10,800)	31,000 (10,600)	30,700 (5020)
9	32,300 (2680) 35,200 (49,600)	32,200 (4410) 35,500 (53,800)	34,500 (24,100) 37,700 (92,400)
10	39,100 (28,300)	39,100 (28,900)	
11	40,800 (14,700) 42,500 (53,800)	40,800 (17,700) 42,600 (64,400)	44,200 (110,000)

^1^ Molar absorption coefficients (dm^3^·mol^–1^·cm^–1^) are in parentheses.

**Table 9:** Angular overlap model simulation assuming the high-spin state.

Parameter/Term	1	2	3
*B*	804	867	966
*C*	3500	3500	2940
*e_σ_*_,av_^1^	5790	5960	5200
^6^ *A*_1_	0	0	0
^2^ *T*_2_	9410	9870	9370
^4^ *T*_1_	11,000	12,100	10,900
^4^ *T*_2_	16,400	16,100	17,200
^2^ *A*_2_	23,100	28,400	21,600
^2^ *T*_1_	23,400	28,500	22,000
^2^ *T*_2_	25,100	30,100	23,800
^4^ *A*_1_	25,600	30,900	24,400
^2^ *E*	27,000	32,000	25,600
^4^ *T*_2_	28,300	32,700	28,000
^4^ *E*	31,200	35,000	31,100
^2^ *T*_1_	32,000	35,600	31,300

^1^ The π orbital effect was not considered.

**Table 10:** Angular overlap model simulation assuming the low-spin state.

Parameter/Term	1	2	3
*B*	739	547	864
*C*	3830	5220 ^2^	3390
*e_σ_*_,av_^1^	9440	11400	8980
^2^ *T*_2_	0	0	0
^6^ *A*_1_	9410	9870	9370
^4^ *T*_1_	11,000	12,100	10,900
^4^ *T*_2_	16,400	16,100	17,200
^2^ *A*_2_	24,000	28,900	22,800
^2^ *T*_1_	24,300	29,100	23,100
^2^ *T*_2_	26,200	30,700	25,300

^1^ The π orbital effect was not considered. ^2^ The value is abnormal compared with the free-ion value.

**Table 11:** Crystallographic data and refinement parameters of **1**, **2**, and **3**.

Complex	1	2	3
Empirical formula	C_40_H_32_Cl_2_F_12_Fe_2_N_8_OP_2_	C_41.8_H_35.4_F_12_Fe_2_N_10.6_O_7.2_P_2_	C_50_H_36_C_l2_F_12_Fe_2_N_9_O_1.5_P_2_
Formula weight	1113.27	1203.04	1259.42
Crystal system	Monoclinic	Triclinic	Triclinic
Space group	*C*2/*c*	*P* $\bar{1}$	*P* $\bar{1}$
*a*/Å	24.5777(8)	12.7471(2)	12.0156(8)
*b*/Å	9.6680(3)	13.6863(3)	13.0997(8)
*c*/Å	18.2923(5)	14.9623(3)	17.0918(11)
α/°	90	75.554(2)	99.7840(10)
β/°	102.781(3)	78.197(2)	97.4510(10)
γ/°	90	76.516(2)	104.7590(10)
*V*/Å^3^	4238.9(2)	2428.25(9)	2521.1(3)
*Z*	4	2	2
Crystal dimensions/mm	0.125 × 0.074 × 0.045	0.190 × 0.180 × 0.070	0.400 × 0.340 × 0.220
*T*/K	105	102	90
λ/Å	0.71073	0.71073	0.71073
*ρ*_calcd_/g·cm^−3^	1.744	1.645	1.659
*µ*/mm^−1^	0.985	0.771	0.841
*F*(000)	2240	1216	1270
2*θ*_max_/◦	55	57	55
No. of reflections measured	15,350	61,890	15,982
No. of independent reflections	4853 (Rint = 0.0268)	12079 (Rint = 0.0403)	11291 (Rint = 0.0165)
Data/restraints/parameters	4853/0/303	12,079/240/790	11,291/0/783
*R*1 ^1^ [*I* > 2.00 σ(*I*)]	0.0306	0.0534	0.0522
*wR*2 ^2^ (all reflections)	0.0780	0.1609	0.1265
Goodness of fit indicator	1.039	1.064	1.126
Highest peak, deepest hole/e Å^−3^	0.393, −0.334	1.690, −0.634	0.855, −0.496
CCDC deposition number	2052873	2052876	2052878

^1^*R*1 = Σ||Fo| − |Fc||/Σ|Fo|, ^2^ *wR*2 = [Σ(*w*(Fo^2^ − Fc^2^)^2^)/Σ*w*(Fo^2^)^2^]^1/2^.

## Data Availability

The crystallographic data are available from the Cambridge Crystallographic Data Centre (CCDC). Other data not presented in Supplementary Materials are available on request from the corresponding author.

## Supplementary Materials

The following are available online. Figure S1: IR spectra of **1**, Figure S2: IR spectra of **2**, Figure S3: IR spectra of **3**, Figure S4: The *χ*_M_*T versus T* plot for **2**, Figure S5: The *χ*_M_*T versus T* plot for **3**.
