# Supplementary material for: Structure Controlling Factors of Oxido-Bridged Dinuclear Iron(III) Complexes
Source: Molecules. 2021 Feb 8;26(4):897. doi: 10.3390/molecules26040897 (PMC7914995; doi:10.3390/molecules26040897)
Supplement: Supplementary file 1 [file molecules-26-00897-s001.pdf]

Supplementary Materials

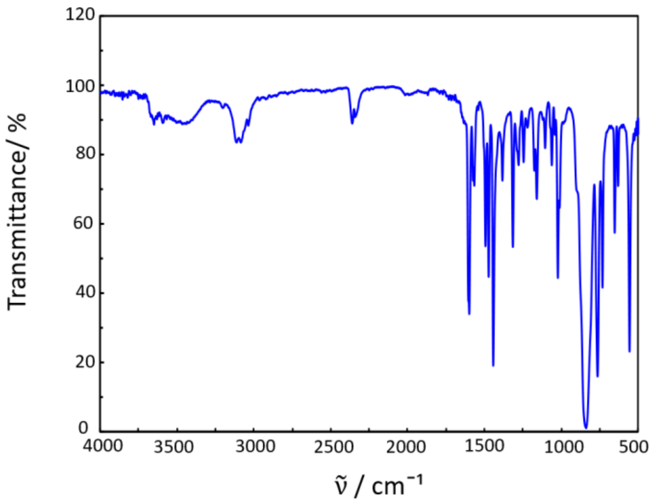

Figure S1. IR spectra of **1**.

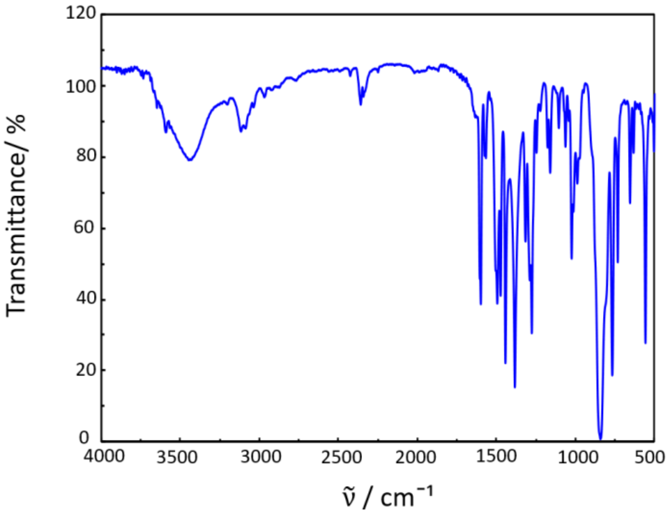

Figure S2. IR spectra of **2**.

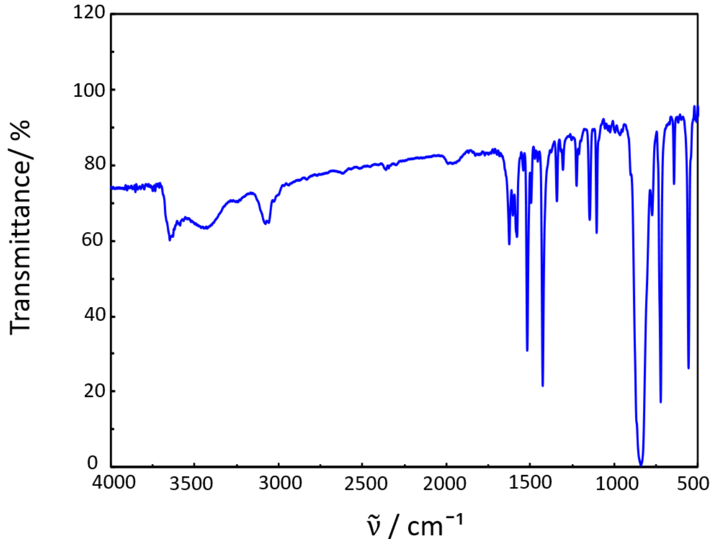

Figure S3. IR spectra of **3**.

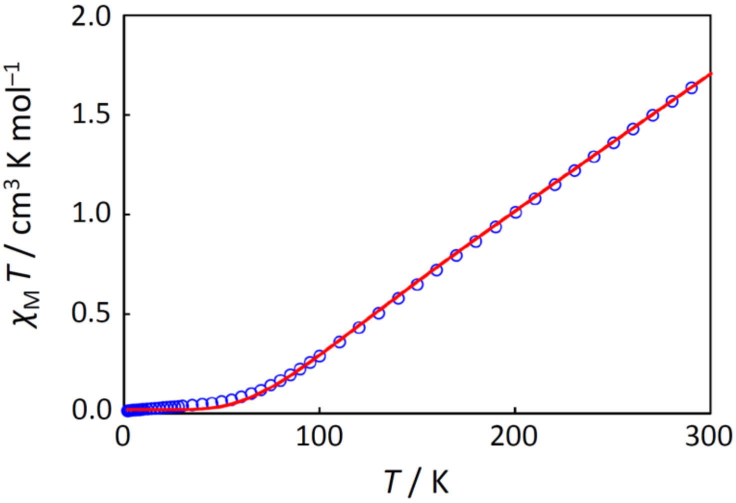

Figure S4. The $\chi_M T$ *versus* T plot for **2** The observed data (○) and the theoretical curve (–).

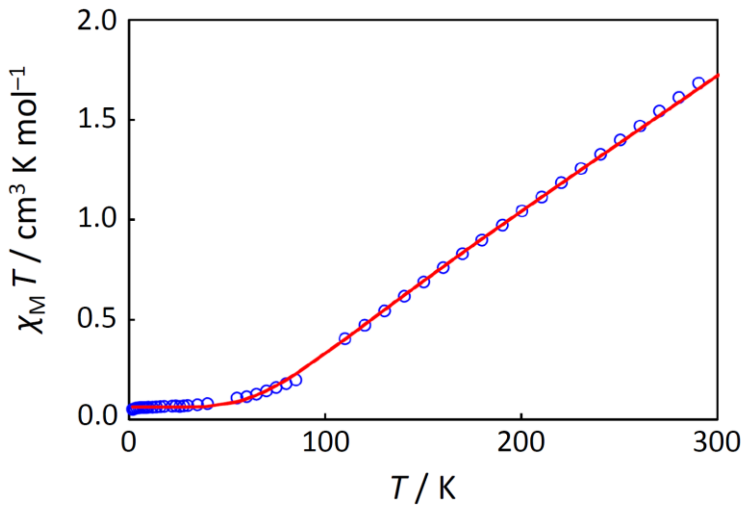

Figure S5. The $\chi_M T$ *versus* T plot for **3** The observed data (○) and the theoretical curve (–).
